# Validation of an automated AI-based micro-CT organ segmentation workflow against expert annotations and its impact on fluorescence quantification

**DOI:** 10.1186/s41747-026-00742-x

**Published:** 2026-05-29

**Authors:** Elena Rama, Sihe Yu, Sarah Schraven, Zachary Gurberg, Ekaterina Savina, Jianjun Liu, Zuzanna Anna Magnuska, Felix Gremse, Fabian Kiessling

**Affiliations:** 1 Institute for Experimental Molecular Imaging, Faculty of Medicine, RWTH Aachen University, Aachen, Germany; 2 Gremse-IT GmbH, Aachen, Germany; 3 Department of Anaesthesia, Intensive Care Medicine and Pain Medicine, Medical University of Vienna, Vienna, Austria

**Keywords:** Artificial intelligence, Fluorescence imaging, Image processing (computer-assisted), Mice, X-ray micro-CT

## Abstract

**Objective:**

Automated, artificial intelligence (AI)-based, organ segmentation has the potential to streamline preclinical imaging workflows, but its suitability must be evaluated not only by geometric accuracy, but also by impact on downstream quantitative analyses. We validated a commercially available AI-based organ segmentation workflow for whole-body micro-computed tomography (micro-CT) data regarding segmentation accuracy, reproducibility, processing time, and effects on fluorescence tomography (FLT) quantification.

**Materials and methods:**

Heart, lungs, liver, and kidneys were segmented in micro-CT scans from 27 mice by three independent experts and by a commercially available automated deep learning tool applied to standard and iteratively reconstructed CT datasets. Segmentation performance was evaluated using the Sørensen-Dice similarity coefficient (DSC) and organ volumes, while downstream effects were assessed using FLT overlay quantification across five fluorescent probes.

**Results:**

AI-based segmentations showed organ-dependent agreement with experts, with lower DSC scores for heart and lungs reflecting systematic boundary differences. Repeated AI segmentations yielded identical results, demonstrating reproducibility while reducing segmentation time from approximately 30 min per mouse to about 5 min, with AI inference completed within seconds. While segmentation geometry and organ volumes differed from expert annotations in an organ-dependent manner, these differences showed no linear relationship with downstream FLT quantification. Iterative CT reconstruction improved the agreement of fluorescence measurements with expert-derived values.

**Conclusion:**

Geometric segmentation metrics alone are insufficient to predict downstream fluorescence quantification, underscoring the need for task-based evaluation strategies in preclinical multimodal imaging. AI-based segmentation offers a reproducible and time-efficient alternative to manual annotation, enabling practical, scalable, and generalizable image analysis.

**Relevance statement:**

Reproducible, AI-based organ segmentation can substantially improve the efficiency and scalability of quantitative preclinical imaging workflows, supporting more robust translation of multimodal imaging biomarkers toward clinical research and future patient-focused applications.

**Key Points:**

- In hybrid micro-CT/FLT imaging, geometric segmentation metrics did not reliably predict downstream fluorescence quantification outcomes.
- AI segmentation assessment should shift from benchmark accuracy metrics toward task-based validation reflecting real downstream imaging applications across different workflows.
- A commercially available AI-based organ segmentation tool demonstrates fully reproducible segmentation and substantially reduced analysis time compared with expert annotations.
- AI applied to iteratively reconstructed CT data improves the agreement of fluorescence quantification with expert annotations compared with standard reconstruction.

**Graphical Abstract:**

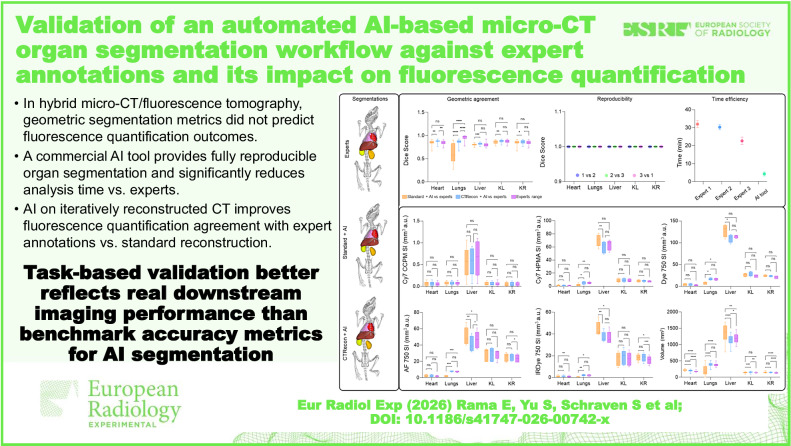

## Background

Medical image segmentation increasingly supports precise and personalized medical decision-making and is becoming integrated into clinical workflows for modalities such as CT and MRI [1]. However, segmentation is equally essential for the quantification of signals in hybrid (nuclear) imaging modalities such as positron emission tomography and single-photon emission CT [2]. In the case of hybrid optical imaging modalities such as fluorescence (FLT) and bioluminescence tomography, segmentation is required for both signal quantification and optical image reconstruction [3, 4].

Micro-computed tomography (micro-CT) is one of the most widely used structural imaging modalities in preclinical research, providing high-resolution anatomical information with homogeneous spatial resolution and fast acquisition times [5]. Owing to its reliability and reproducibility, micro-CT is frequently used both as a standalone modality and as an anatomical reference in multimodal imaging. However, micro-CT suffers from low soft-tissue contrast, limiting accurate segmentation of soft-tissue boundaries. Although contrast agents can enhance soft-tissue visibility, most segmentation tasks still rely on manual delineation, which is time-consuming and user-dependent. As dataset sizes grow, manual segmentation increasingly represents a bottleneck for efficiency, scalability, and reproducibility. These challenges highlight the need for automated and generalizable segmentation solutions for multi-center and longitudinal studies.

Building on this need for automated and reliable segmentation methods, the U-Net model proposed by Ronneberger et al became widely adopted for robust biomedical segmentation, thanks to its symmetric encoder–decoder design and skip connections that preserve fine spatial details [6]. Several variations have been proposed to further enhance U-Net’s feature extraction or generalizability. For example, Oktay et al introduced attention mechanisms to prioritize the foreground features, while suppressing the background noise; Dense U-Net utilized dense connectivity to promote feature reuse and richer representations; U-Net++ refined multi-scale feature fusion; and the nnU-Net helped transition the ‘blueprint’ into the era of large-scale, multi-centric datasets by turning the original U-Net architecture into a self-configuring framework [3, 7–10]. Together, these developments established U-Net-based architectures as a robust and widely adopted foundation for medical image segmentation across different imaging modalities and anatomical targets.

In this study, we address the need for automated, reproducible, and scalable segmentation by evaluating a semantic segmentation workflow implemented in a commercially available software platform (Imalytics Preclinical, Gremse-IT GmbH) that assigns each voxel to a specific organ class, enabling rapid generation of anatomical maps. The workflow is based on a U-Net-based model combined with an approach for consistent image alignment to generate the precise organ delineation essential for quantitative analysis in preclinical small-animal imaging.

To comprehensively assess the performance of our proposed method, we conducted experiments on a dataset comprising scans of 27 mice. We compared CT-based automated organ segmentation results with manual annotations from three independent experts using the Sørensen-Dice coefficient (DSC) and organ-volume metrics. We evaluated two segmentation strategies: (1) manual expert annotations, and (2) deep learning-based automated CT segmentation using a U-Net-based AI model applied to standard reconstructed (standard + AI) and iterative CT reconstructed micro-CT images (CTRecon + AI). Unlike conventional reconstruction approaches that process each sub-scan independently, an iterative reconstruction integrates information from adjacent scans, enabling consistent whole-body reconstruction and reducing discontinuities across regions [11]. This results in more coherent images, facilitating more robust subsequent organ segmentation. We performed fluorescence overlay analyses to examine how each segmentation method influences downstream optical signal quantification.

## Methods

### Datasets

To evaluate the automated segmentation approach, a dataset of whole-body three-dimensional scans acquired from 27 athymic nude ATHYM-Foxn1nu/nu and BALB/cAnNRj mice (Janvier Labs) was used. These scans were obtained from previous studies, in which all imaging data had already been collected and ethically approved [12, 13].

### Micro-CT and FLT imaging

Each imaging session consisted of FLT and micro-CT acquisitions. FLT scans were performed at multiple projection angles on a hybrid micro-CT-optical imaging system (MILabs B.V.) using excitation and emission wavelength settings appropriate for the respective fluorescent probes (Table 1). Following FLT imaging, whole-body micro-CT scans were acquired in normal scan (tube voltage/current: 55 kVp and 0.17 mA or 65 kV and 0.13 mA; isotropic voxel size: 140 µm).

**Table 1:** Fluorescent probes used for FLT imaging and corresponding spectral properties

Fluorescent probe	Excitation maximum (nm)	Emission maximum (nm)
Cy7 CCPM	730	775
AF750 HPMA	749	775
IRDye750 HPMA	756	776
Cy7 HPMA	750	773
DY750	747	776

*AF750 HPMA* Alexa Fluor 750 N-(2-Hydroxypropyl) methacrylamide, *Cy7 CCPM* Cy7 core-crosslinked polymeric micelles, *Cy7 HPMA* Cy7 N-(2-Hydroxypropyl) methacrylamide, *DY750* DyLight750, *IRDye 750 HPMA* Infrared Fluorescent Dye 750 N-(2-Hydroxypropyl) methacrylamide

### Image reconstruction

Micro-CT–FLT fusion was achieved using the fixed pattern of 44 air-filled reference markers embedded in the multimodal mouse holder (MILabs B.V.), which ensured stable spatial registration between both modalities.

#### FLT reconstruction

FLT image reconstruction was performed as previously described by Gremse et al [14]. Briefly, fluorescence distributions were reconstructed using a model-based approach incorporating the absorption and scattering maps derived from the micro-CT data. The reconstruction relied on a normalized Born approximation and non-negative least squares minimization with regularization to ensure stable solutions, resulting in quantitative three-dimensional fluorescence volumes. The FLT volumes were reconstructed with an isotropic voxel spacing of 280 µm.

#### Standard CT reconstruction

Standard micro-CT images and projection data were reconstructed using the MILabs reconstruction software with a default analytical reconstruction workflow based on filtered back projection, producing isotropic whole-body CT volumes with a voxel size of 140 µm.

#### CT iterative reconstruction

CT reconstruction was performed using the CTRecon 1.1.1.8 software package (Gremse-IT GmbH) employing a model-based iterative reconstruction approach, as previously described [11]. Briefly, the reconstruction of the micro-CT volumes was performed using the Adam iterative algorithm with the same isotropic voxel size of 140 µm and 30 iterations, ensuring direct comparability with the standard reconstruction. Unlike analytical reconstruction methods such as filtered back projection, iterative reconstruction improves the image through multiple steps. Sequential reconstruction of sub-scans ensured continuity across the full field of view and reduced boundary artifacts between adjacent scan regions. A binning factor of 2 was applied during projection preprocessing, and the reconstructed volumes were exported as 32-bit voxel data.

### Segmentation by three independent experts

Three independent experts were selected to perform organ segmentations, representing different levels of experience (> 4 years, ~1 year, and < 1 year, respectively). Heart, lungs, liver, and kidneys, which were subsequently differentiated into kidney right (KR) and kidney left (KL), were selected for segmentation and subsequent analysis. All expert annotations were performed using Imalytics Preclinical 3.1.2 (Gremse-IT GmbH) following a standardized segmentation protocol [15]. Briefly, lungs were semi-automatically segmented by applying a threshold function below a certain value, -600 Hounsfield units, followed by manual removal of the trachea using a dedicated segmentation class. All other organs were delineated manually.

### Deep learning-based automated segmentation

Automated organ segmentation was performed using the new integrated AI mouse mode in Imalytics Preclinical (version 3.1.6; Gremse-IT GmbH.). This mouse mode applies a deep learning-based inference model to an aligned input image to generate segmentation regions. Gremse-IT’s mouse organ segmentation model was used to obtain the segmentation of the heart, lungs, liver, and kidneys from micro-CT images.

This model was trained on 872 micro-CT scans of mice acquired from multiple institutions and imaging systems from six different vendors (*e.g*., MILabs, Molecubes, and Mediso), resulting in variability in scanner platforms and acquisition settings. The 27 scans analyzed in the present study originated from two separate studies and were not part of the training dataset. These scans were therefore used exclusively for independent evaluation of the model performance. All scans were manually annotated by domain experts using tools provided by Imalytics Preclinical. Prior to training, scans were aligned to a common anatomy-based atlas space, intensity-normalized, then resampled to uniform voxel dimensions. This preprocessing enforces consistent spatial alignment across the dataset, allowing the model to remain efficient in both parameter count and computational cost. The anatomy-based alignment additionally enables the extraction of spatial feature maps used as model inputs.

The model architecture is based on the U-Net implementation from the open-source library MONAI (available at: https://project-monai.github.io/) with four layers of depth of channel dimension 128, 256, 512, and 1,024, respectively, in addition to two residual units. A combination of online and offline data augmentations was used to improve the robustness of the model. An AdamW optimizer and compound loss function were utilized, resulting in a final > 93% DSC score for each region.

To assess the influence of CT reconstruction strategy on automated segmentation performance, the same AI model was applied to standard (standard + AI) and iteratively reconstructed (CTRecon + AI) micro-CT images. The resulting segmentations were resampled to the native space by the mouse mode and visualized in the software, ready for subsequent quantitative analysis.

### Sørensen–Dice similarity coefficient (DSC)

To assess the agreement between the manual annotations from the three independent experts, the segmentations generated by the standard + AI model, and the CTRecon + AI method, we computed the DSC using Imalytics Preclinical. This similarity coefficient quantifies the spatial overlap between two segmentations, A and B, and ranges from 0 to 1, indicating no overlap or perfect agreement, respectively. It is defined as:

$${DSC}=\frac{2\left|A\cap B\right|}{\left|A\right|+\,\left|B\right|}$$

where ∣A∣ and ∣B∣ represent the number of voxels in each segmentation, and ∣ A ∩ B ∣ denotes the number of voxels common to both. As reported in the literature, DSC scores above 0.8 were ranked good, ≤ 0.8 to 0.6 as medium, and < 0.6 as poor [4, 16].

To establish a reference for evaluation, we quantified inter-expert variability by calculating the DSC scores for every pairwise combination of the three independent experts (*e.g*., Expert 1 *versus* Expert 2; Expert 1 *versus* Expert 3; *etc*.). DSC scores between automated segmentations and the expert annotations were then computed for each organ and mouse.

### Overlay analysis

To evaluate the downstream impact of the standard + AI and CTRecon + AI methods on optical signal quantification, fluorescence overlay analyses were performed. Total signal intensity normalized by organ volume (mm³·a.u.) was used as the best representing value to capture differences between expert- and model-derived segmentations. To assess potential systematic under- or over-segmentation, organ volumes (mm³) were also analyzed.

### Statistics

Statistical analyses were performed using the GraphPad Prism 9 software (GraphPad Software). Normality of the data distribution was assessed using the Shapiro–Wilk test. Differences in DSC scores, fluorescence overlay values, and organ volumes between segmentation methods (experts, standard + AI, and CTRecon + AI) were evaluated using a mixed-effects model with segmentation method and organ as fixed effects and mouse as a repeated factor. *Post hoc* multiple comparisons between methods within each organ were performed using Tukey correction. Data are expressed as mean ± standard deviation. Statistical significance was considered for values *p* < 0.05.

## Results

### DSC score

To establish a reproducible and scalable segmentation pipeline for preclinical multimodal imaging, we first evaluated whether automated organ segmentation using a U-Net-based AI tool, applied to standard and CTRecon-processed micro-CT data, could achieve accuracy comparable to expert manual segmentations. For this purpose, we calculated the DSC scores for all pairwise expert comparisons as well as for the standard + AI and CTRecon + AI approaches (Table 2).

**Table 2:** DSC scores showing the inter-expert agreement and the performance of the standard + AI and CTRecon + AI segmentation methods for each organ

	Organs				
	Heart	Lungs	Liver	KL	KR
Expert 1 *versus* 2	0.85	0.94	0.81	0.84	0.82
Expert 1 *versus* 3	0.87	0.92	0.78	0.85	0.83
Expert 2 *versus* 3	0.89	0.98	0.80	0.88	0.88
Standard + AI *versus* Expert 1	0.81	0.61	0.76	0.82	0.79
Standard + AI *versus* Expert 2	0.89	0.61	0.83	0.87	0.86
Standard + AI *versus* Expert 3	0.85	0.61	0.80	0.86	0.86
CTRecon + AI *versus* Expert 1	0.84	0.85	0.79	0.84	0.81
CTRecon + AI *versus* Expert 2	0.90	0.85	0.87	0.89	0.91
CTRecon + AI *versus* Expert 3	0.88	0.85	0.81	0.88	0.88

*CTRecon* + *AI* Iterative CT reconstructed micro-CT images, *KL* Kidney left, *KR* Kidney right, *Standard* + *AI* Standard reconstructed micro-CT images

Inter-expert DSC scores were consistently above the 0.8 threshold typically classified as good, with the exception of only one value (0.78) for the liver, indicating slightly lower agreement between experts.

**Fig. 1:**
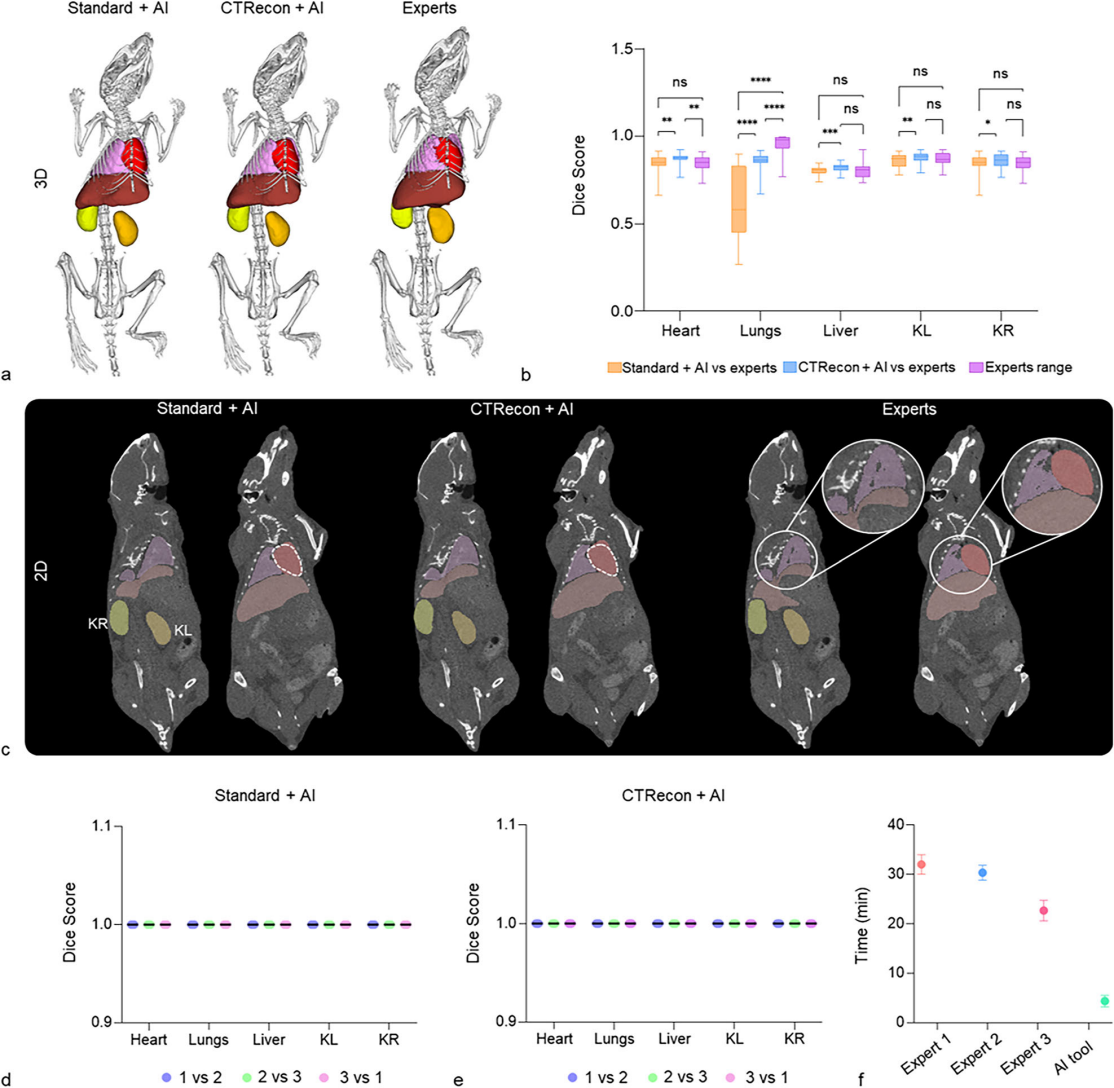
Organ segmentation and DSC score analysis. **a** Representative three-dimensional organ segmentations are shown for one mouse for the three independent experts, the standard + AI, and the CTRecon + AI method. **b** DSC score analysis illustrates the similarity between each expert and the standard + AI and CTRecon + AI approach. DSC values are therefore reported as “method *versus* expert,” reflecting pairwise comparisons between the segmentation method (standard + AI or CTRecon + AI) and each expert segmentation. DSC scores for the heart obtained with the standard + AI approach were comparable to expert annotations, whereas the CTRecon + AI method showed a small but statistically significant deviation from expert agreement. In contrast, lung segmentations yielded significantly lower DSC scores for both automated approaches compared with expert annotations. For the liver and both kidneys, DSC scores obtained with the standard + AI and CTRecon + AI approaches were comparable to expert agreement levels. **c** Representative coronal 2D micro-CT slices overlaid with segmentation masks illustrate qualitative differences between the three experts, standard + AI and CTRecon + AI segmentation methods, highlighting variations in heart boundary definition and lung coverage (heart: white dotted outlines; lungs: magnified insets). **d**, **e** DSC score analysis of triplicate segmentations generated with the standard + AI and CTRecon + AI workflows shows identical results, highlighting the reproducibility of the AI-based segmentation tool. **f** The AI-based segmentation was approximately 7.5-fold faster than manual segmentation. Statistical significance is indicated as follows: ns = *p* ≥ 0.05, * *p* < 0.05, ** *p* < 0.01, *** *p* < 0.001, and **** *p* < 0.0001. CTRecon + AI, Iterative CT reconstructed micro-CT images; KL, Kidney left; KR, Kidney right; Standard + AI, Standard reconstructed micro-CT images

DSC scores obtained for the automated segmentation approaches were generally lower than the expert-expert values. While most organs remained within the range typically considered moderate agreement, lung segmentation using the standard + AI approach showed substantially lower DSC scores (0.61), indicating borderline poor agreement compared with expert annotations. As shown in Fig. 1a, b, DSC scores for the heart obtained using the standard + AI approach were comparable to expert annotations, whereas the CTRecon + AI method showed a statistically significant deviation from expert agreement (*p* = 0.0098, 95% confidence interval (CI) of the mean DSC difference: 0.007–0.055). In contrast, for the lungs, both standard + AI and CTRecon + AI yielded DSC scores that were significantly lower than those observed between expert annotations (standard + AI *versus* experts: *p* < 0.0001, 95% CI of the mean DSC difference: -0.447 to -0.220; CTRecon + AI *versus* experts: *p* < 0.0001, 95% CI of the mean DSC difference: -0.134 to -0.065). For liver and kidney segmentation, DSC scores obtained with the standard + AI and CTRecon + AI approaches were comparable to expert agreement levels. Interestingly, no statistically significant improvement in DSC scores was observed when using the CTRecon + AI method compared with the standard + AI approach, indicating that differences in CT reconstruction strategy did not substantially affect geometric segmentation agreement. This suggests that DSC score performance in this workflow is primarily driven by the AI model’s atlas alignment and learned spatial priors rather than by reconstruction-dependent image texture or voxel intensity differences.

**Fig. 2:**
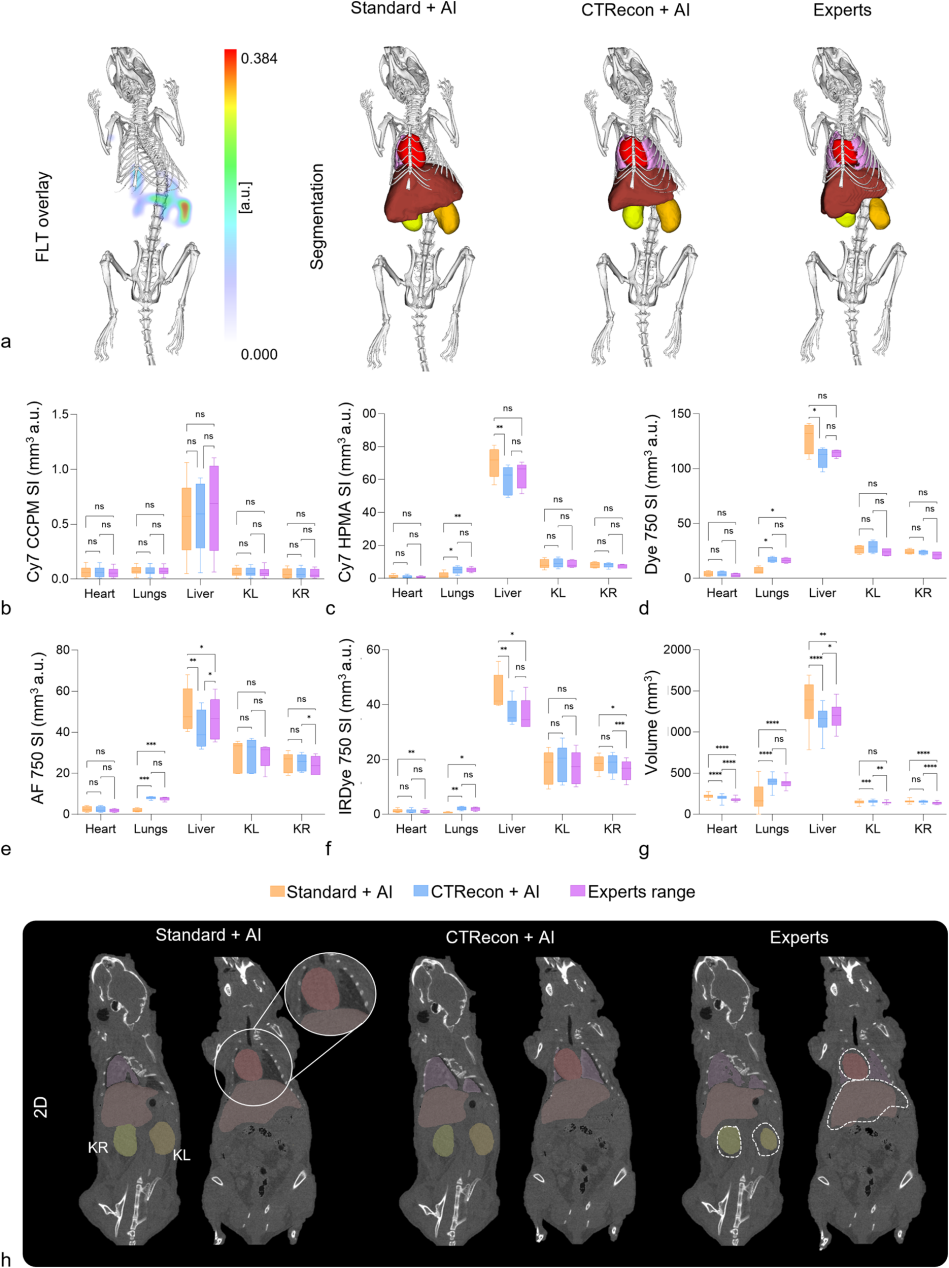
Fluorescence tomography reconstruction and quantification. **a** Representative three-dimensional organ segmentations and corresponding fluorescence overlays are shown for the three experts, the standard + AI, and the CTRecon + AI model. **b**–**f** Fluorescence overlay values, expressed as total fluorescence signal normalized by organ volume (mm³·a.u.), are shown separately for each fluorescent probe. Differences between automated and expert segmentations occur in an organ- and probe-dependent manner for both standard + AI and CTRecon + AI approaches, with the lungs exhibiting the highest sensitivity to segmentation differences, while fluorescence quantification of other organs remains comparatively robust. **g**, **h** Volume analysis (mm³) and two-dimensional micro-CT coronal slices demonstrate a statistically significant overestimation of heart and KR volume by both standard + AI and CTRecon + AI approaches, while lung volumes were significantly under-segmented by the standard + AI method but remained comparable to expert annotations with CTRecon + AI. Liver volumes showed opposite trends between the two automated approaches, being oversegmented by the standard + AI workflow and slightly under-segmented by CTRecon + AI. Additionally, only CTRecon + AI produced significant over-segmentation of the left kidney (heart, liver, KL, and KR: white dotted outlines; lungs: magnified insets). Statistical significance is indicated as follows: ns = *p* ≥ 0.05, * *p* < 0.05, ** *p* < 0.01, *** *p* < 0.001, and **** *p* < 0.0001. AF750 HPMA, Alexa Fluor 750 N-(2-Hydroxypropyl) methacrylamide; Cy7 CCPM, Cy7 core-crosslinked polymeric micelles; Cy7 HPMA, Cy7 N-(2-Hydroxypropyl) methacrylamide; DY750, DyLight750; IRDye750, Infrared Fluorescent Dye 750 N-(2-Hydroxypropyl) methacrylamide; CTRecon + AI, Iterative CT reconstructed micro-CT images; FLT, Fluorescence tomography; KL, Kidney left; KR, Kidney right; Standard + AI, Standard reconstructed micro-CT images; SI, Signal intensity

Two-dimensional micro-CT slices overlaid with the segmentations from the standard + AI, CTRecon + AI, and one representative expert annotation allow direct qualitative visual comparison of segmentation differences, especially for the lungs and kidneys (Fig. 1c). For the heart, qualitative inspection revealed differences in boundary definition between automated and expert segmentations. While DSC score analysis indicated a statistically significant deviation only for the CTRecon + AI approach, visual comparison suggested that both standard + AI and CTRecon +AI produced similar heart segmentations that differed from expert annotations. For the lungs, the three independent experts generated segmentations using a threshold-based approach, which primarily segments the aerated lung volume and therefore tends to exclude radiopaque structures such as intrapulmonary vessels and tissue components. In contrast, both standard + AI and CTRecon + AI were trained to segment the entire lung region, including both air-filled spaces and surrounding tissue. Consequently, the automated segmentations produced smoother and more spatially continuous lung volumes that included regions not captured by the expert masks. These qualitative observations are further examined and quantitatively assessed in the subsequent volume and fluorescence overlay analyses (Fig. 2).

To assess the reproducibility of organ segmentations obtained using the integrated Imalytics AI tool, the model was applied three times to all micro-CT volumes in the dataset, including both standard reconstructed and iteratively reconstructed images. Repeated application of the AI-based segmentation tool to the same datasets resulted in identical segmentation masks across all runs, yielding DSC scores equal to 1.0 for all organs for both standard + AI and CTRecon + AI (Fig. 1d, e). This indicates perfect overlap and deterministic behavior and extremely high reproducibility of the automated approach.

Beyond its reproducibility, the AI-based organ segmentation approach offers a substantial practical advantage in terms of processing time, requiring only minimal user interaction to generate the final organ segmentations (Fig. 1f) (Expert 1: 32 ± 1.63 min; Expert 2: 30 ± 1.24 min; Expert 3: 28 ± 1.69 s; AI tool: 4 ± 0.83 min).

### Overlay analysis

After determining that the standard + AI and CTRecon + AI approaches showed only minor deviations from the performance of human segmentations, we next assessed the reliability of the complete segmentation workflow and evaluated how each method influenced fluorescence quantification. To this end, we performed fluorescence overlay analyses, with the resulting values reported in Table 3 for each fluorescent probe, independent expert, standard + AI, and CTRecon + AI.

**Table 3:** Total fluorescence intensity normalized by organ volume (mm³·a.u.), reported for each fluorescent probe and organ, comparing the three independent experts with the standard + AI and CTRecon + AI segmentation methods

Probe	Method	Heart	Lungs	Liver	KL	KR
Cy7 CCPM	Experts	0.06 ± 0.01	0.07 ± 0.01	0.62 ± 0.06	0.06 ± 0.01	0.04 ± 0.01
	Standard + AI	0.06 ± 0.00	0.07 ± 0.00	0.54 ± 0.00	0.06 ± 0.00	0.05 ± 0.00
	CTRecon + AI	0.06 ± 0.00	0.07 ± 0.00	0.57 ± 0.00	0.06 ± 0.00	0.05 ± 0.00
Cy7 HPMA	Experts	0.51 ± 0.21	4.22 ± 1.23	51.37 ± 14.58	6.99 ± 2.27	5.60 ± 1.76
	Standard + AI	1.00 ± 0.00	1.77 ± 0.00	70.47 ± 0.00	8.77 ± 0.00	8.11 ± 0.00
	CTRecon + AI	0.89 ± 0.00	5.06 ± 0.00	59.80 ± 0.00	9.22 ± 0.00	8.09 ± 0.00
Dye 750	Experts	2.76 ± 0.56	16.36 ± 0.08	114.17 ± 3.34	23.87 ± 1.83	21.11 ± 1.46
	Standard + AI	4.10 ± 0.00	6.78 ± 0.00	128.51 ± 0.00	26.71 ± 0.00	24.17 ± 0.00
	CTRecon + AI	3.93 ± 0.00	16.98 ± 0.00	110.48 ± 0.00	28.76 ± 0.00	23.86 ± 0.00
AF750 HPMA	Experts	1.87 ± 0.11	7.67 ± 0.02	46.34 ± 1.89	28.84 ± 1.01	23.79 ± 0.56
	Standard + AI	2.52 ± 0.00	2.03 ± 0.00	50.71 ± 0.00	28.51 ± 0.00	25.15 ± 0.00
	CTRecon + AI	2.46 ± 0.00	8.09 ± 0.00	41.39 ± 0.00	29.08 ± 0.00	25.17 ± 0.00
IRDye 750	Experts	0.96 ± 0.06	2.01 ± 0.08	36.29 ± 0.49	16.92 ± 1.18	16.09 ± 1.21
	Standard + AI	1.21 ± 0.00	0.63 ± 0.00	44.59 ± 0.00	17.17 ± 0.00	18.49 ± 0.00
	CTRecon + AI	1.08 ± 0.00	2.18 ± 0.00	37.16 ± 0.00	18.50 ± 0.00	18.41 ± 0.00

Data are given as mean ± standard deviation

*AF750 HPMA* Alexa Fluor 750 N-(2-Hydroxypropyl) methacrylamide, *Cy7 CCPM* Cy7 core-crosslinked polymeric micelles, *Cy7 HPMA* Cy7 N-(2-Hydroxypropyl) methacrylamide, *DY750* DyLight750, *IRDye750* Infrared Fluorescent Dye 750 N-(2-Hydroxypropyl) methacrylamide, *CTRecon* + *AI* Iterative CT reconstructed micro-CT images, *KL* Kidney left, *KR* Kidney right, *Standard* + *AI* Standard reconstructed micro-CT images

Fluorescence overlay analyses were performed separately for each of the five fluorescent probes to account for probe-dependent biodistribution patterns (Fig. 2a, f). Across all fluorescent probes, apart from Cy7 core-crosslinked polymeric micelles (CCPM), the lungs emerged as the organ most sensitive to segmentation differences, whereas fluorescence quantification for the heart, liver, and kidneys was generally more robust and probe dependent. Importantly, differences between standard + AI and CTRecon + AI followed a probe- and organ-dependent pattern. However, across multiple fluorescent probes, the CTRecon + AI approach generally showed fewer statistically significant deviations from the expert-based analysis, suggesting that iterative reconstruction can partially improve downstream quantification.

This pattern was partially reflected in the volume analysis, as shown by the box plots (Fig. 2g) and representative 2D micro-CT slices overlaid with the corresponding organ segmentations (Fig. 2h). Volume measurements revealed organ-specific segmentation biases. Both standard + AI and CTRecon + AI approaches significantly oversegmented heart and KR compared with expert annotations (heart: standard + AI *versus* experts: *p* < 0.0001, 95% CI of the mean volume difference: 32.10–53.35; CTRecon + AI *versus* experts: *p* < 0.0001, 95% CI of the mean volume difference: 15.36–34.60; KR: standard + AI *versus* experts: *p* < 0.0001, 95% CI of the mean volume difference: 10.10–29.11; CTRecon + AI *versus* experts: *p* < 0.0001, 95% CI of the mean volume difference: 8.810–23.05). In contrast, lung volumes were significantly under-segmented by the standard + AI method (*p* < 0.0001, 95% CI of the mean volume difference: -226.0 to -102.6), whereas CTRecon + AI volumes remained comparable to expert annotations. Liver volumes showed opposite trends between the two automated approaches, being significantly oversegmented by the standard + AI method (*p* = 0.005, 95% CI of the mean volume difference: 41.05–251.9) but slightly under-segmented by CTRecon + AI (*p* = 0.042, 95% CI of the mean volume difference: -105.6 to -1.810). For KL, only the CTRecon + AI segmentation produced significantly larger volumes than the expert annotations (*p* = 0.011, 95% CI of the mean volume difference: 5.190–21.44), indicating over-segmentation (Table 4). Although segmentation-related volumetric differences contributed to organ-dependent variability, they did not consistently translate into changes in fluorescence quantification, highlighting the complex and organ-specific relationship between segmentation geometry and quantitative optical imaging.

**Table 4:** Organ volume (mm³·a.u.) reported for organ, comparing the three independent experts with the standard + AI and CTRecon + AI segmentation methods

	Organs				
	Heart	Lungs	Liver	KL	KR
Experts	179.15 ± 14.67	383.22 ± 19.51	1,202.12 ± 26.32	140.81 ± 8.79	136.91 ± 12.11
Standard + AI	221.88 ± 0.00	218.93 ± 0.00	1,348.58 ± 0.00	148.54 ± 0.00	156.52 ± 0.00
CTRecon + AI	204.14 ± 0.00	391.74 ± 0.00	1,148.43 ± 0.00	154.12 ± 0.00	152.84 ± 0.00

Data are given as mean ± standard deviation

*CTRecon* + *AI* Iterative CT reconstructed micro-CT images, *KL* Kidney left, *KR* Kidney right, *Standard* + *AI* Standard reconstructed micro-CT images

## Discussion

In this study, we evaluated the performance of an AI-based organ segmentation approach integrated in Imalytics Preclinical by comparing it with manual annotations from three independent experts in a preclinical multimodal imaging setting. Using DSC score analysis, volume measurements, and downstream fluorescence quantification, we assessed not only geometric agreement but also the practical impact of segmentation differences on quantitative optical imaging. Our results demonstrate that the AI-based segmentation does not uniformly match expert-level geometric accuracy across all organs. Nevertheless, the method yields fully reproducible segmentations and, despite organ- and probe-dependent differences, generally results in fluorescence quantification that remains within or close to the expert-defined reference ranges, indicating that deviations in geometric agreement do not systematically translate into compromised downstream fluorescence readouts.

Rather than relying on a single manual annotation as ground truth, we evaluated segmentation performance in the context of inter-observer variability among three independent experts [17, 18]. As frequently reported, manual organ segmentation remains influenced by the operator’s experience, interpretation of anatomical boundaries, and image quality, contributing to inter-observer variability [19, 20]. This was reflected by the variability observed among our three experts, particularly for anatomically complex or low-contrast structures. Considering this inherent variability, AI-based tools are not assessed with the aim of surpassing expert performance, but rather of operating within human variability, with the key advantage of providing fully reproducible results.

As previously shown, DSC scores are valuable for assessing the initial performance of a segmentation method in terms of geometric agreement, but they do not directly reflect how segmentation differences influence performance in the quantitative imaging task for which the data are acquired [4, 21]. In our study, probe-resolved fluorescence overlay analyses demonstrated that differences in geometric agreement and organ volume between expert and AI-based segmentations propagated to fluorescence quantification in an organ- and probe-dependent manner. The lungs emerged as the organ most sensitive to segmentation differences, exhibiting statistically significant deviations in fluorescence signal for several fluorescent probes, which can be attributed to their low intrinsic soft-tissue contrast in micro-CT, presence of air-tissue interfaces, as well as fine vascular and airway structures. In contrast, fluorescence quantification for the heart, liver, and kidneys was generally more robust despite segmentation-related differences in geometric overlap and organ volume. These findings confirm that geometric overlap metrics alone are insufficient to predict quantitative accuracy and emphasize the importance of task-based evaluation strategies. For example, to overcome such issues, Liu et al and Mueller et al proposed standardized task-specific evaluations as a mandatory means for translating AI segmentation into practice, rather than relying on region-overlap alone [21, 22]. In line with these observations, our findings suggest that validation of AI-based segmentation methods may benefit from moving beyond purely benchmark accuracy metrics toward task-based frameworks that assess their impact within the full imaging workflow. Such considerations are likely relevant beyond the specific micro-CT/FLT setting investigated here and may extend to other imaging modalities and quantitative imaging pipelines in which segmentation represents an intermediate step for downstream analysis.

At the same time, segmentation accuracy exhibited organ-dependent behavior [23]. Qualitative inspection (Fig. 1d) showed organ-dependent boundary differences, particularly for the lungs, where inter-expert disagreement was also evident. Quantitative volume analysis confirmed statistically significant segmentation biases for specific organs, most prominently for the heart, lungs, and KR, and to a lesser extent for the liver and KL. Importantly, however, the impact of these volumetric differences on fluorescence quantification was not uniform, varying across organs and fluorescent probes (Fig. 2b, f). These results highlight the importance of evaluating automated segmentation methods also on an organ-specific basis and in relation to their impact on quantitative imaging outcomes. Complementary reliability metrics, such as intraclass correlation coefficients, may further support this assessment in future studies.

As previous studies in clinical imaging have shown that iterative reconstruction can influence automated post-processing workflows and quantitative image analysis by reducing noise and altering image characteristics, we further investigated whether this approach could also enhance automated segmentation performance in micro-CT imaging [24, 25]. We observed that iterative reconstruction altered voxel intensities and image texture, thereby influencing downstream fluorescence quantification without substantially changing segmentation geometry. While both standard + AI and CTRecon + AI exhibited organ-dependent volumetric biases relative to expert annotations, *i.e*., overestimation of the heart and KR, under-segmentation of the lungs by the standard + AI approach, opposite trends for the liver, and over-segmentation of the left kidney by CTRecon + AI, the impact of these biases on fluorescence quantification differed between reconstruction strategies. Particularly, fluorescence overlay analysis showed that segmentations derived from CTRecon-processed images generally yielded fluorescence values closer to the expert annotations than those obtained with standard reconstruction. This indicates that iterative reconstruction primarily mitigates the quantitative consequences of segmentation biases rather than correcting segmentation geometry, which remains largely governed by learned spatial context and atlas-based alignment. Importantly, volumetric differences did not consistently translate into proportional changes in fluorescence quantification, highlighting a complex non-linear relationship between segmentation geometry, reconstruction characteristics, and downstream optical signal analysis. Future studies should therefore systematically investigate how reconstruction algorithms and parameter settings (*e.g*., different iterative schemes or analytical reconstructions) influence segmentation-derived volumes and downstream fluorescence quantification, including whether these effects depend on organ-specific signal distributions and fluorescent probe biodistribution patterns.

The AI segmentation tool integrated in Imalytics Preclinical demonstrated complete reproducibility and a substantial reduction in analysis time. Repeated application of the AI tool yielded identical segmentation results, in contrast to the inter-expert variability observed with manual annotations, indicating robust and systematic behavior of the automated AI-based approach. Importantly, the total processing time, including loading the datasets, adjusting the anatomical atlas, and generating the AI-based organ segmentations, was approximately 5 min per mouse, while the AI inference step required only a few seconds. In comparison, manual organ segmentation required an average of 30 min per mouse, depending on user experience and organ complexity. This combination of reproducibility and time efficiency enhances scalability, reduces user burden, and supports the application of automated segmentation in large-scale, longitudinal, and high-throughput preclinical imaging studies.

However, some limitations of this study should be acknowledged. Our evaluation focused on a single, commercially available AI segmentation tool integrated within one software platform, and the dataset was acquired using a single imaging system and acquisition protocol. Domain shifts related to scanner vendors, imaging sites, and acquisition protocols can influence deep learning segmentation performance, particularly when models are applied to unseen domains [26, 27]. In addition, the AI segmentation relies on a predefined anatomical atlas with limited flexibility, which does not fully account for constrained animal positioning. In preclinical multimodal imaging, mice are often firmly restrained during anesthesia to minimize motion and enable accurate CT-FLT coregistration, resulting in slight but systematic deviations from the atlas positioning that may contribute to segmentation inaccuracies for certain organs. Future work will compare AI-based segmentation tools, assess robustness across acquisition protocols and imaging platforms, and explore approaches to improve tolerance to constrained animal positioning.

In conclusion, our findings demonstrate that AI-based organ segmentation tools offer a reproducible and time-efficient alternative to manual annotation for preclinical multimodal imaging workflows. Importantly, DSC scores and volumetric agreement did not reliably predict downstream fluorescence quantification, demonstrating that geometric accuracy alone is insufficient. Together, these results support the practical utility of AI-based segmentation for accelerating and standardizing quantitative preclinical imaging analyses, while underscoring the importance of task-based evaluation when assessing segmentation performance for translational imaging applications and, ultimately, clinical decision-making.

## Data Availability

The datasets used and/or analyzed during the current study are available from the corresponding author on reasonable request.

## Abbreviations

AI: Artificial intelligence
CI: Confidence interval
CT: Computed tomography
CTRecon + AI: AI model applied to iterative reconstructed micro-CT images
DSC: Sørensen–Dice similarity coefficient
FLT: Fluorescence tomography
KL: Kidney left
KR: Kidney right
Micro-CT: Micro-computed tomography
Standard + AI: AI model applied to standard reconstructed micro-CT images
